# Suicidal intention, psychosocial factors and referral to further treatment: A one-year cross-sectional study of self-poisoning

**DOI:** 10.1186/1471-244X-10-58

**Published:** 2010-07-26

**Authors:** Mari A Bjornaas, Knut E Hovda, Fridtjof Heyerdahl, Karina Skog, Per Drottning, Anders Opdahl, Dag Jacobsen, Oivind Ekeberg

**Affiliations:** 1 Department of Acute Medicine, Oslo University Hospital Ulleval, N-0407 Oslo, Norway; 2 Department of Behavioural Sciences in Medicine, University of Oslo, N-0317 Oslo, Norway; 3 Department of Medicine, Oslo University Hospital Aker, N-0514 Oslo, Norway; 4 Department of Medicine, Lovisenberg Hospital, N-0165 Oslo, Norway; 5 Department of Medicine, Diakonhjemmet Hospital, N-0319 Oslo, Norway

## Abstract

**Background:**

Patients treated for self-poisoning have an increased risk of death, both by natural and unnatural causes. The follow-up of these patients is therefore of great importance. The aim of this study was to explore the differences in psychosocial factors and referrals to follow-up among self-poisoning patients according to their evaluated intention.

**Methods:**

A cross-sectional multicenter study of all 908 admissions to hospital because of self-poisoning in Oslo during one year was completed. Fifty-four percent were females, and the median age was 36 years. The patients were grouped according to evaluated intention: suicide attempts (moderate to high suicide intent), appeals (low suicide intent) and substance-use related poisonings. Multinomial regression analyses compared patients based on their evaluated intention; suicide attempts were used as the reference.

**Results:**

Of all self-poisoning incidents, 37% were suicide attempts, 26% were appeals and 38% were related to substance use. Fifty-five percent of the patients reported previous suicide attempts, 58% reported previous or current psychiatric treatment and 32% reported daily substance use. Overall, patients treated for self-poisoning showed a lack of social integration. Only 33% were employed, 34% were married or cohabiting and 53% were living alone. Those in the suicide attempt and appeal groups had more previous suicide attempts and reported more psychiatric treatment than those with poisoning related to substance use. One third of all patients with substance use-related poisoning reported previous suicide attempts, and one third of suicide attempt patients reported daily substance use. Gender distribution was the only statistically significant difference between the appeal patients and suicide attempt patients. Almost one in every five patients was discharged without any plans for follow-up: 36% of patients with substance use-related poisoning and 5% of suicide attempt patients. Thirty-eight percent of all suicide attempt patients were admitted to a psychiatric ward. Only 10% of patients with substance use-related poisoning were referred to substance abuse treatment.

**Conclusions:**

All patients had several risk factors for suicidal behavior. There were only minor differences between suicide attempt patients and appeal patients. If the self-poisoning was evaluated as related to substance use, the patient was often discharged without plans for follow-up.

## Background

Long-term mortality after self-poisoning, by both natural and unnatural causes [1], is much higher than for the general population, irrespective of intention [2]. In a 20-year follow-up study of self-poisoning in Oslo, male gender, lower social group, drug abuse, and lower level of consciousness were all independent predictors of death. Suicidal intention was not an independent predictor of death in general, but it was the only independent predictor of later suicide. For suicide attempt patients, both sociodemographic and psychiatric factors are associated with later suicide [3,4]. For those who have not made suicide attempts, there is less literature, although the risk of death both in general and by suicide is increased for substance use disorders [5].

One would expect suicide attempt patients to differ from those who have not attempted suicide in more than the evaluated intention, even among self-poisoning cases. However, recent research indicates that the populations overlap, with repetitions of self-poisoning during the same year differing in their evaluated intentions [6]. Therefore, more information about patients treated for self-poisoning, even if they have not attempted suicide, is needed.

The majority of studies in the field focus on subgroups of self-poisoning, and use terms such as medically serious suicide attempters [7], those who deliberately self-harm [8], and those with nonfatal drug overdoses [9]. The challenge behind this classification is the correct evaluation of the intention. Patients who present at emergency departments with self-poisoning are often comatose, and immediate evaluation is difficult. Furthermore, they can be reluctant to report the use of illegal substances. In suicide attempt patients, the wish to die may vary over time, which can further complicate the evaluation of intention. Different approaches in follow-up may therefore be necessary for substance use-related poisoning and suicide attempt patients. It is unclear whether the morbidity of the substance users treated in emergency departments has been underestimated [10]. The risk of further suicidal behavior in patients reporting suicidal intention has led to psychiatric follow-up of these patients; being referred for specialist follow-up reduces the risk of a repeat attempt [11]. Suicide attempters who suffer from substance use disorders are less likely to receive psychiatric follow-up [12]. More information about the follow-up of these patients is needed, irrespective of intention.

Accordingly, the aims of this study on patients who presented with self-poisoning in emergency departments in Oslo during a one-year period were to study: 1) the evaluation of intention, made by both patients and physicians; 2) the sociodemographic and psychiatric characteristics of these patients; 3) how these characteristics vary according to the evaluated intention of self-poisoning patients; and 4) the plans for follow-up of these patients at discharge.

## Methods

### Design of the study

This cross-sectional multicenter study was performed from April 1, 2003 until March 31, 2004 and involved all four Oslo hospitals that treat patients with self-poisoning, together with pediatric departments, the Oslo Emergency Ward (outpatient clinic), the ambulance service, and the Institute of Forensic Medicine. This was done to obtain a complete one-year picture of all patients contacting health care services because of self-poisoning in the capital of Norway.

This paper presents data from all hospitalized adults in Oslo regarding evaluation of intention, psychiatric history, sociodemographic variables and referral to follow-up. Clinical and epidemiological data have been presented separately [13].

The inclusion criteria for the present part of the study were exposure to a drug or another agent in toxic amounts leading to hospital admission in adults (≥ 16 years). Exclusion criteria were chronic poisoning and patients with other primary diagnoses, such as pneumonia, even if there was an additional self-poisoning. However, if the self-poisoning would have required medical attention, the case was included. All cases considered to be accidental nonself-poisoning were excluded from further analyses, including carbon monoxide poisoning caused by fire accidents (n = 13), taking prescribed medication in incorrect doses due to lack of understanding (n = 24), and forced intake or accidental poisoning (n = 2). The study population included 908 admissions, of which 54% were females. The median age was 36 years (range = 16-89 years). The population of Oslo in 2003 was 521,886, of whom 428,198 were > 16 years, which gives an annual incidence of 0.21%

### Data collection

Physicians obtained data by completing a standardized registration form as soon as the patient was ready for an interview. Verbal informed consent was obtained. For patients who did not regain consciousness or had cerebral damage (n = 16), data were obtained from medical files. Only one patient refused to participate.

### Criteria for classifications

Physicians' evaluations of the reasons for poisoning were based on all information available, including patients' own reported intentions. Three categories were used: suicide attempt (possible or definite), appeal and substance use-related poisoning. Suicide attempt patients were those evaluated by the treating physician as having a moderate to high suicide intent. Appeal patients were those with low or no suicide intent. In these cases, the self-poisoning could not be classified as a substance use-related poisoning or as a suicide attempt, as suicidal intent was low or nonexistent. Patients with substance use-related poisoning had used substances of abuse (ethanol, opiates or opioids, gamma-hydroxybutyrate (GHB), amphetamines, ecstasy, cocaine, benzodiazepines or cannabis, or a combination of substances) in a way that led to hospitalization, and where the intended purpose was thought to be recreational use. The distinction between the three categories was not necessarily clear cut, but the physicians were asked to categorize each patient into one of the groups based on their best clinical judgment. To separate suicide attempts from appeals, special attention was given to letters that confirmed suicidal intent, supposed lethal doses of the toxic agent or other active procedures designed to ensure a lethal outcome. Information from other sources, such as ambulance personnel and companions, was also considered. Accidental poisonings that were not self-inflicted were excluded from further analyses (n = 39).

For patients' own evaluations of intent, five different categories were used: intention to die, to escape from problems, to make an impact on personal relationships, substance use-related poisoning and unknown. Only one category was chosen for each patient. Subsequently, these answers were grouped into the following categories to compare the patient's and the physician's evaluated intention: suicide attempt (intention to die), appeal (to escape from problems and to make an impact on personal relationships) and substance use-related poisoning. Cases where the patient's evaluated intention was unknown were excluded from the studies of agreement.

Sociodemographic variables recorded were marital status, living conditions, country of origin according to place of birth or parental place of birth, occupational status and education (highest level completed according to the Norwegian education system).

Previous suicide attempts and psychiatric treatment (both current and former) were recorded, as reported by patients. For former psychiatric treatment, the highest level of treatment was used in further analyses; for example, psychiatric ward admission was rated higher than psychiatric outpatient treatment. Patients were also asked to report the frequency of their substance use and what kind of substances they were using.

Referrals to further follow-up services at the time of discharge were recorded. The categories were: referral to a General Practitioner, a suicide prevention team, substance abuse treatment, a psychiatric outpatient clinic, a psychiatric ward (voluntarily or involuntarily), other arrangements and no plans for aftercare. More than one category could be recorded for each patient. Some patients left the hospital against medical advice, and they were treated as a separate group in further analyses.

### Statistics

The standardized registration forms were optically scanned and processed using TeleForm Desktop version 9.1 (TeleForm, Verity Inc., Sunnyvale, California). Statistics were performed using SPSS, version 16.0 (SPSS Inc., Chicago, Illinois). Cohen's Kappa was used to compare the doctor's and the patient's evaluation of intention. Multinomial regression analyses were used to compare the groups according to psychosocial factors, with the doctor's evaluation of intention (suicide attempt, appeal, substance use-related poisoning) as the dependent variable. Suicide attempt, as assessed by physicians, was used as the reference category. Crude and adjusted ORs were computed, with a 95% confidence interval. Only variables with a significant crude value (*p *≤ 0.02) were included in the multinomial analyses. Variables where only the proportion of unknown answers was significantly different between the groups were excluded from the multinomial analyses.

### Ethics

Treatment was given in accordance with the standard hospital protocols, and the study was done in accordance with the Helsinki Declaration. Permission was obtained from The National Data Inspectorate and the Regional Ethics Committee. The links between patients' names and social security numbers and the study case numbers were stored by Statistics Norway.

## Results

### Intention

Of the 908 admissions, 10% were evaluated by the physician as definite suicide attempts and 26% as possible suicide attempts (Table 1). All patients evaluated as definite suicide attempts stated a wish to die. However, 5% of those eventually evaluated as appeal patients also stated a wish to die. In total, 36% were evaluated as suicide attempt patients and 26% as appeal patients. Substance use-related poisoning was seen in 38% of cases, of whom 59% also stated substance use as the reason for the self-poisoning.

**Table 1:** Assessment of intention by physicians and patients

Physician's evaluation of intention	Patient's evaluation of intention					
	
	Intention to die n (%)	To escape from problems n (%)	To make an impact on personal relationships n (%)	Substance use-related poisoning n (%)	Unknown n (%)	Total n (%)
**Definite suicide attempt**	91 (34)	-	-	-	1 (1)	92(10)
**Possible suicide attempt**	130 (48)	56 (33)	16 (24)	6 (3)	32 (17)	240 (26)
**Appeal**	46 (17)	95 (56)	50 (77)	4 (2)	37 (20)	232 (26)
**Substance use-related poisoning**	4 (1)	20 (12)	-	202 (95)	118 (63)	344 (38)

**Total**	271 (100)	171 (100)	66 (100)	212 (100)	188 (100)	908 (100)

When the patients evaluated their intention, 30% stated a wish to die, 23% stated substance use as the reason for the admission and 19% wanted to escape from problems. In the appeal group, 11% wanted to escape from problems and 6% wanted to make an impact on personal relationships.

The overall agreement between the physician's and the patient's evaluation of intention was high when patients' answers were grouped in the three main categories: suicide attempts, appeal and substance use-related poisoning. The agreement had a Kappa value of 0.68.

### Sociodemographic characteristics

There were more females in the suicide attempt (63%) and appeal groups (72%), whereas males dominated the substance use-related poisoning group (65%) (Table 2). Males were more likely than females to be evaluated as having substance use-related poisoning than attempting suicide: OR 3.16 (95% C.I., 2.27-4.41) (Table 3).

**Table 2:** Sociodemographic characteristics of patients treated for self-poisoning in Oslo over one year, according to intention

	Suicide attempt n = 332	Appeal n = 232	Substance use-related poisoning n = 344	Total n = 908
**Male gender**	37%	28%	65%	45%

**Age**				
16-29	27%	34%	37%	33%
30-49	52%	47%	39%	46%
≥ 50	21%	19%	24%	22%

**Country of origin**				
Norway	81%	82%	88%	84%
Other European country	5%	6%	5%	6%
Asian country	10%	11%	4%	8%
Other	4%	0.01%	3%	3%
Unknown	n = 1	n = 3	n = 6	n = 10

**Living conditions**				
Living alone	51%	53%	55%	53%
With parents	7%	11%	15%	11%
With spouse/others	37%	31%	27%	32%
In institution	5%	5%	3%	4%
Unknown	n = 67	n = 40	n = 94	n = 201

**Occupational status**				
Employee/student	30%	36%	34%	33%
Sick leave	16%	11%	6%	11%
Unemployed	12%	13%	23%	16%
Retired	7%	3%	11%	7%
Permanent disability	35%	37%	27%	32%
Other/unknown	n = 54	n = 44	n = 79	n = 177

Note: The percentages are calculated for each column, rather than for each row.

**Table 3:** Comparison of sociodemographic characteristics in patients treated for self-poisoning in Oslo, according to intention

	Substance use-related poisoning vs. suicide attempt				
	
	Crude			Adjusted	
	
	*p*	OR	95% CI	OR	95% CI
**Male gender**	< 0.001	3.13*	2.29-4.29	3.16**	2.27-4.41

**Age**	0.007				
16-29		ref			
30-49		0.53**	0.38-0.76	0.51*	0.34-0.77
≥ 50		0.80	0.52-1.21	0.87	0.50-1.53

**Country of origin**	0.002				
Norway		ref			
Other European country		0.91	0.46-1.78	0.72	0.35-1.47
Asian country		0.34*	0.17-0.67	0.23**	0.11-0.49
Other		0.83	0.36-1.91	0.71	0.28-1.75
Unknown		5.43	0.65-45.43	5.21	0.59-46.0

**Occupational status**	< 0.001				
Employee/student		ref			
Sick leave		0.31**	0.16-0.59	0.30**	0.15-0.61
Unemployed		1.83*	1.09-3.08	1.65	0.95-2.86
Retired		1.32	0.69-2.52	1.13	0.51-2.50
Permanent disability		0.70	0.46-1.07	0.73	0.45-1.20
Other/unknown		1.38	0.87-2.18	1.11	0.67-1.83

**Living conditions**	0.003				
Living alone		ref			
Living with parents		2.10*	1.14-3.85	2.21*	1.12-4.37
Living with others		0.68	0.46-1.00	0.74	0.49-1.13
In institution		0.61	0.25-1.52	0.74	0.28-1.95
Other/unknown		0.79	0.50-1.26	1.40	0.90-2.17

*p < 0.05

**p < 0.001

Suicide attempt was used as the reference category. Only variables with a significant crude value (*p *≤ 0.02) were included in the multinomial analyses. Variables where only the proportion of unknown answers was significantly different between the groups were excluded. The appeal group did not differ from the suicide attempt group in any respect other than gender in the multinomial analyses, and therefore the figures are not included here.

Males were less likely to be evaluated as appeal patients than females: adjusted OR 0.63 (95% C.I., 0.43-0.92). There were no other statistically significant differences in sociodemographic variables between the appeal group and the suicide attempt group in the multinomial analyses.

There were, however, several significant differences between the suicide attempt patients and those with substance use-related poisoning. Patients who were 30-49 years old were less likely to be in the substance use-related group than those who were younger, compared with suicide attempt patients: OR 0.51 (95% C.I., 0.34-0.77) (Table 3).

The majority of the patients (84%) were originally from Norway. Immigrants from Asia were less likely to be in the substance use-related poisoning group than in the suicide attempt group, compared with native Norwegians: OR 0.23 (95% C.I., 0.11-0.49).

Fifty-three percent of all patients were living alone. Even when age differences were corrected for, those who were living with their parents were more likely to be in the substance use-related poisoning group than in the suicide attempt group, compared with those living alone: OR 2.21 (95% C.I., 1.12-4.37).

Thirty-three percent of the patients were employees/students, 32% were permanently disabled and 16% were unemployed. Those on sick leave were less likely to be in the substance use-related poisoning group than in the suicide attempt group, compared with employees: OR 0.30 (95% C.I., 0.15-0.61).

Overall, 34% were married or cohabiting, 19% were divorced, 4% were widows/widowers and 42% had never been married. Thirty-eight percent of all patients had only completed the minimum nine years of primary and secondary school required by law. There were no statistically significant differences between the three groups according to marital status or educational status.

### Psychiatric characteristics

Previous suicide attempts were reported by 55% of all patients: 68% of the suicide attempt group, 62% of the appeal group and 32% of the substance use-related poisoning group (Table 4). There were no statistically significant differences between the appeal group and the suicide attempt group. However, even when age was corrected for, those who reported previous suicide attempts were less likely to be in the substance use-related poisoning group than in the suicide attempt group, compared with those without such an attempt: OR 0.33 (95% C.I., 0.22-0.49) (Table 5).

**Table 4:** Psychiatric characteristics of self-poisoning, according to intention

	Suicide attempt n = 332 (%)	Appeal n = 232 (%)	Substance use-related poisoning n = 344 (%)	Total n = 908 (%)
**Previous suicide attempt**				
Yes	68%	62%	32%	55%
None	32%	38%	68%	45%
Unknown	n = 38	n = 19	n = 110	n = 167

**Previous psychiatric treatment**†				
Yes, outpatient clinic	15%	23%	21%	19%
Yes, psychiatric ward	42%	35%	19%	33%
None	43%	41%	60%	48%
Unknown	n = 36	n = 23	n = 86	n = 145

**Current psychiatric treatment**				
Yes, outpatient clinic	44%	40%	18%	34%
Yes, psychiatric ward	9%	9%	3%	7%
None	47%	51%	79%	59%
Unknown	n = 29	n = 19	n = 87	n = 135

Note: The percentages are calculated for each column, rather than for each row.

† Highest level of treatment registered.

**Table 5:** Comparison of psychiatric characteristics of patients treated for self-poisoning in Oslo, according to intention

	Substance use-related poisoning vs. suicide attempt				
	
	Crude			Adjusted	
	*p*	OR	95% CI	OR	95% CI
**Male gender**	< 0.001	3.13**	2.29-4.29	2.81**	1.99-3.97
**Previous suicide attempt**	< 0.001				
No		ref		ref	
Yes		0.23**	0.16-0.33	0.33**	0.22-0.49
Unknown		1.72*	1.10-2.70	1.62	0.97-2.73
**Psychiatric treatment**†	< 0.001				
No		ref		ref	
Yes		0.27**	0.18-0.39	0.42**	0.27-0.63
Unknown		1.21	0.74-1.97	0.84	0.47-1.49

† Psychiatric treatment includes both current and previous treatment.

*p < 0.05

**p < 0.001

Suicide attempt was used as the reference category. Only variables with a significant crude value (*p *≤ 0.02) were included in the multinomial analyses. Variables where only the proportion of unknown answers was significantly different between the groups were excluded. Age was adjusted for. The appeal group did not differ from the suicide attempt group in any respect other than gender in the multinomial analyses, and therefore the figures are not included here.

At the time of admission, 41% of patients were having current/ongoing psychiatric treatment, 7% as inpatients. A total of 33% had previously been psychiatric inpatients: 42% of the suicide attempt group, 35% of the appeal group and 19% of the substance use-related poisoning group. Only 31% of self-poisoning patients had never received psychiatric treatment. Those who reported any psychiatric treatment were less likely to be in the substance use-related poisoning group than in the suicide attempt group: OR 0.33 (95% C.I., 0.22-0.49). There was no significant difference in the level of psychiatric treatment between the appeal and suicide attempt groups.

Gender and age were included in the multinomial analyses of psychiatric factors, but did not alter the main findings.

### Substance use

Daily substance use was reported by 37% of all patients, while 11% reported that they never used such substances.

Of those in the substance use-related poisoning group, 48% reported daily substance use, while for those in the appeal and suicide attempt groups the figures were 25% and 35%, respectively. Those who reported daily substance use were more likely to be in the substance use-related poisoning group: OR 5.57 (95% C.I., 2.63-11.79). There was no significant difference in substance use between the appeal and suicide attempt groups.

In the suicide attempt group, daily use of alcohol was reported by 18%, while 19% used prescription drugs daily. Opioids and amphetamines were used weekly or more frequently by 6%.

In the appeal group, 13% reported daily use of alcohol and 14% reported daily use of prescription drugs. Daily use of opioids was reported by 4%.

In the substance use-related poisoning group, 25% reported daily alcohol use and 23% reported daily use of prescription drugs. Thirteen percent used opioids on a daily basis, with another 4% using these less often. Eight percent used GHB weekly or more often, while 13% used amphetamines with the same frequency. Cocaine was used by 7% of substance use-related poisoning patients in total.

### Referral to follow-up

Of all patients, 18% were discharged without plans for further treatment or follow-up: 36% of those with substance use-related poisoning, 10% of appeal patients and 5% of suicide attempt patients. Those who were discharged without plans for follow-up were more likely to be in the substance use-related poisoning group than those who received follow-up: OR 11.0 (6.35-19.02). Only 10% of substance use-related poisoning patients had plans for follow-up from substance abuse treatment services, while 28% were discharged with plans for follow-up by their general practitioner only.

Those who received any psychiatric follow-up were more likely to be in the suicide attempt group than those who did not receive such treatment--irrespective of level of treatment (Table 6). Of those in the substance use-related poisoning group, 10% were referred to a psychiatric outpatient clinic, 0.6% to a psychiatric ward voluntarily and 3% involuntarily. More of those evaluated as appeal patients (47%) were referred to psychiatric outpatient treatment than those evaluated as suicide attempt patients (39%). However, 18% of suicide attempt patients were admitted voluntarily to a psychiatric ward, and 20% were admitted involuntarily.

**Table 6:** Referral to follow-up for patients treated for self-poisoning

		Compared with suicide attempts		
		
Referral to follow-up OR (95% C.I.)	Suicide attempt	Appeal	Substance use-related poisoning	Total
	**%**	**%** **OR (95% C.I)**	**%** **OR (95% C.I)**	**%**

No referral	5% ref	10% 2.28* (1.18-4.39)	36% 11.0 ** (6.35-19.02)	18%
General practitioner	19% ref	31% 1.96* (1.32-2.90)	28% 1.69* (1.17-2.42)	25%
Suicide prevention team	11% ref	10% 0.98 (0.57-1.70)	2% 0.18** (0.08-0.40)	7%
Substance abuse treatment	4% ref	7% 1.70 (0.79-3.64)	10% 2.69* (1.39-5.20)	7%
Psychiatric outpatient clinic	39% ref	47% 1.14* (1.00-1.98)	11% 0.19** (0.13-0.29)	30%
Psychiatric ward, voluntary	18% ref	9% 0.44* (0.26-0.75)	1% 0.03** (0.01-0.11)	9%
Psychiatric ward, involuntary	20% ref	6% 0.25**(0.14-0.46)	3% 0.13** (0.07-0.13)	10%
Other referral	9% ref	7% 0.70 (0.37-1.33)	17% 2.08 * (1.31-3.33)	11%
Left hospital against medical advice	2% ref	0.1% 0.24 (0.03-1.97)	6% 3.18* (1.25-8.06)	3%

*p < 0.05

**p < 0.001

More than one category was possible for each patient. In the multinomial analyses, each group is compared with the suicide attempt group, which is therefore listed as the reference category.

Of all patients, 3% left hospital against medical advice, 6% of those in the substance use-related poisoning group and 2% of suicide attempt patients. These were not included in the figures for no referral. Those who left hospital against medical advice were more likely to be in the substance use-related poisoning group: OR 3.18 (1.25-8.06).

## Discussion

Self-poisoning patients had several psychosocial risk factors for suicidal behavior. There were only minor differences between suicide attempt patients and appeal patients; the only significant difference between these groups was a higher percentage of females among appeal patients. Substance use-related poisoning patients differed from suicide attempt patients in some respects, but displayed several risk factors for suicidal behavior as well. Overall, more than half of the patients reported previous suicide attempts, and 58% reported previous or current psychiatric treatment. Daily substance use was reported by one third of all patients. Furthermore, one third was listed as permanently disabled, and one third had only completed the lowest mandatory level of education. In this context, 18% of the patients were discharged without plans for follow-up. Patients with substance use-related poisoning were less likely to be provided with follow-up plans for discharge; 36% were discharged without such plans. If intention is given too much weight, a large group of substance use-related poisoning patients seems likely to be excluded from further aftercare, despite their well-known risk of increased mortality in general and of suicide in particular.

Females were more likely to be evaluated as appeal patients, but in all other respects, there were no differences between these patients and those evaluated as suicide attempt patients. The gradient of suicidal intent affects the risk of completing suicide in the long term, as medically serious suicide attempts are at higher risk and use different substances [14,15]. The higher proportion of suicide attempt patients undergoing current psychiatric treatment may imply that, at the time of the self-poisoning, the patients had a higher burden of psychiatric illness, although the difference was not statistically significant.

There were several differences in psychosocial risk factors between substance use-related poisoning patients and suicide attempt patients. Males younger than 30 years old, who were living with their parents or who reported substance use of any frequency, were more likely to be evaluated as substance use-related poisoning patients than suicide attempt patients. Being from Asia, being temporarily on sick leave or reporting previous suicide attempts and/or psychiatric treatment reduced the likelihood of the episode being evaluated as a substance use-related poisoning. The gender difference corresponds with other studies, with more males among substance users [16] and more females among suicide attempt patients, although being male is a risk factor for completing suicide [17]. The age difference is also supported by other studies [14]. Being from Asia reduced the likelihood of the episode being evaluated as a substance use-related poisoning. In Oslo, the largest group of Asian immigrants is from Pakistan (3.7% of all inhabitants), and Islam is the dominant religion. Differences in substance use may be explained partly by religious beliefs [18], but information about this subgroup is scarce. Only 6% of patients with substance use-related poisonings reported to be on sick leave, and therefore those on sick leave were less likely to be evaluated as substance use-related poisoning patients than suicide attempt patients. This may be related to a higher proportion of those in the substance use-related poisoning group stating they were unemployed, although this was not statistically significant.

Almost one in five patients was discharged without any plans for follow-up, even when those who left hospital against medical advice were excluded from the analysis. Although different approaches are probably needed for suicide attempt patients and substance use-related poisonings, the number of patients discharged without follow-up seems too large in the context of increased mortality and suicide risk in this patient group [2]. Of those in the substance use-related poisoning group, more than one third was discharged without follow-up. Only 10% had plans for substance use treatment. It could be argued that many of these patients did not want further follow-up, or at least not the kind of follow-up that they were offered. However, the proportion of patients who reported daily substance use, previous suicide attempts and psychiatric treatment indicates that these patients were in need of follow-up as well. Of all self-poisonings in Oslo during the study period (2997 poisoning episodes treated by health care services), 69% were treated outside the hospital by ambulance services, or in the Oslo Emergency Ward (an outpatient clinic) [19]. Only 31% were transferred to hospital. Those who were not transferred to higher levels of care were more often poisoned by drug and alcohol abuse than were those who were hospitalized. In Oslo, the majority of opiate or opioid poisonings are treated at the scene by the ambulance services, unlike many other countries [20]. Routinely, all patients are offered the opportunity to be taken to the outpatient clinic or to the hospital, but most patients refuse this. It is, however, alarming that the great majority of patients with substance use-related poisonings never reach hospital for a more thorough evaluation of their intention and their medical and social needs, and hence do not receive a plan for follow-up. Furthermore, among those who are treated at hospital, more than one third are discharged without plans for follow-up.

The difference in follow-up according to intention corresponds to previous studies, which found that suicide attempt patients suffering from substance use disorders were less likely to receive psychiatric follow-up [12]. Suicide attempt patients were admitted to psychiatric ward treatment in 38% of cases, 20% involuntarily. In a Swedish study from 1994, 57% of suicide attempters were admitted to psychiatric inpatient care, but since then, outpatient care has been used more extensively in all health care services [21]. Still, 5% of suicide attempters were discharged without plans for follow-up, despite their well-known risk of further suicidal behavior, especially in the short term [22]. According to guidelines, they should have been assessed, but some leave hospital before assessment, mainly during holidays, weekends and nights. Only 10% of those in the substance use-related poisoning group were referred to substance use treatment. Although the treatment need may vary within this subgroup of patients, the low percentage that were referred to substance use treatment was particularly low in this study, and lower than in a study from Switzerland where 33% of opioid addicts treated for acute overdose were referred to further follow-up [23]. However, there are few studies on the follow-up of patients treated for self-poisoning, even for the subgroups, and we do not know enough about the effectiveness of the treatment offered regarding mortality and suicide risk.

The patient's intention was evaluated by both patient and physician in each self-poisoning episode, and the overall agreement was good. The physician knew the patient's evaluation at the time he or she evaluated the patients, and the variables were therefore not independent, as evaluation of intention is always based partly on the patient's reported intention. One third of all patients were evaluated as suicide attempt patients, and the importance of recognizing these patients is demonstrated by the increased risk of suicide completion among suicide attempters [24]. Previous suicide attempts were reported by more than half of all self-poisoning patients. A previous suicide attempt is the strongest known predictor of completing suicide [22], and the high proportion of such acts among self-poisoning patients is therefore alarming. Those evaluated as suicide attempt patients *at present *were more likely to receive a higher level of care at discharge than those evaluated as appeal patients. Both suicide attempts and appeals are aspects of suicidal behavior [25]. Among repeaters of self-poisoning, intention has been shown to vary between different admissions during the same year [6]. The proportion of risk factors for suicidal behavior among substance use-related poisoning patients and the extent of substance use among suicide attempt patients may explain some of these findings. If the intention of a current episode is given too much weight, physicians may underestimate the risk of suicide in the long term among those evaluated as nonsuicidal, especially among the appeal patients.

A study of self-poisoning patients in Oslo in 1980 found that being evaluated as a suicide attempt patient was not an independent predictor of death in general [2], although other studies have found increased mortality among suicide attempters compared with the general population [3]. This highlights the fact that the risk of death is also increased among self-poisoning patients who are evaluated as appeal and substance use-related poisoning patients [2]. Therefore, patient characteristics other than intention alone may explain this increased mortality.

One third of the patients were employed, one third were married or cohabiting, and half of them were living alone. Compared with the general population of Oslo [26], where unemployment was 2.2% in 2003, 16% of patients treated for self-poisoning were unemployed. Only 6.6% of the general population, but 11% of the patients, were on sick leave. Among the general population, 48% were married or cohabiting, whereas this was true for only 34% of the patients. Among the general population, 16% had completed only the minimum level of education, whereas 38% of patients treated for self-poisonings had completed only the minimum education. Lack of social integration has been identified in previous studies of suicide attempters and is thought to be an important risk factor for suicidal behavior [27]. The low level of education, lack of association with the labor market and high proportion of being single found here among self-poisoning patients was similar to that found in studies on suicide attempters [28]. In a recent cross-national study on suicide attempters, the same picture was seen, with the exception of employment status, which did not appear as a risk factor for suicidal behavior [29]. However, in the present study, these risk factors were found even among substance use-related poisoning patients and appeal patients. Lack of social integration has been found to be a risk factor for increased mortality even among samples of healthy employees [30], and the lack of social integration may therefore partly explain the increased mortality observed among self-poisoning patients, as well as the increased suicide risk, irrespective of intention [2].

There was considerable substance use among patients treated for self-poisoning, as one third reported daily substance use. More substance use was reported by those evaluated as substance use-related poisoning patients, but even among those evaluated as suicide attempt patients, 32% reported daily substance use. In a study of self-poisoning patients from 2001, nine out of ten patients had traces of drugs of abuse in their blood or urine samples [31]. The present figures may therefore be considered a minimum. Ethanol and prescribed medications such as benzodiazepines were most commonly reported, which corresponds to the most common toxic agents seen in the actual self-poisoning episodes in this study population [13]. The availability of these substances was therefore important, both for daily use and in the actual self-poisoning episode. Substance use is the second most frequent psychiatric precursor to suicide, exceeded only by depressive disorders [32]. The increased mortality found among substance users is also well known [5]. The high proportion of daily substance use seen in this study may therefore partly explain the increased mortality of self-poisoning patients, even in patients who have not made suicide attempts.

### Strength and limitations

All medical departments in Oslo were included over one year to minimize selection bias and to facilitate comparison of the study sample to a well-defined background population. Whether or not all eligible patients were included can always be questioned when so many co-workers are involved, but careful follow-up of the participating departments throughout the study period was done to minimize the number of missed cases. However, the complete multicenter study, which the present study was part of, included patients at three levels of healthcare (ambulance services, the outpatient clinic and hospitals), and transfers between these levels were common. Because of each patient's unique social security number, we were able to trace all patients through different levels of health care. This helped to make the study more complete because each patient could have been included in up to three treatment facilities during each episode, and a study of repetition patterns among the patients revealed that very few patients were lost to follow-up when transferred to a higher level [19]. In each hospital, a study coordinator supervised the inclusion of patients, and the study group supervised these coordinators on a weekly basis to ensure a high participation rate. We believe that our figures reflect the actual number of poisoning episodes as closely as was possible.

No validated scales or forms were used in the evaluation of intention, and this might be seen as a possible limitation of this study. However, our method resembles the evaluations done in emergency departments every day, and it is therefore easier to generalize to clinical practice. The validity of self-reported psychosocial factors may also be questioned, but in general, the information obtained in this study matches what is available in the clinical setting. The form was based on clinical terms commonly used in clinical interviews and in the patient's charts. We therefore believe it to be as reliable and valid as any clinical evaluation, with its strengths and weaknesses.

Not all forms were complete, but in most cases, the percentage of 'not known' responses was less than 10%, with the exception of educational status, which was 32%, and the patients' reported frequency of substance use, which was 21%. Overall, 'not known' responses were more common in the substance use-related poisoning group, which was probably related to the shorter duration of stay for these patients. This is also a possible reason for the limited knowledge about this patient group in previous studies.

The field of suicidology suffers from lack of consistency in the terms used [33]. Clinically, there is a spectrum of self-poisonings varying from the clearly planned, medically serious suicide attempt with an outspoken intention to die, to impulsive actions that are never life threatening and where the intention is not to die but is, perhaps, to make an appeal to others [25]. The term 'appeal' is problematic, as some fear that it implies a devaluation of the patient's intention or that doctors will take these actions, and therefore these patients, less seriously. Although both groups show aspects of suicidal behavior, the classification of all these cases as suicide attempts may be seen as an oversimplification. Many appeal patients wished for changes, such as achieving relational or social solutions. In other cases, patients wanted to escape from an unbearable situation by going to sleep or reducing inner tension. Even though there is no way for the physician to prove that a wish to die was never present, the patients engaged in acts that they definitely knew were not life threatening. However, we lack an appropriate term for this group of patients. The terms "gesture" and "cry of pain" patients have been used in the past, but are now seen as even less appropriate. In this study, the term "appeal" was used for lack of a better term and because this term was used in the original study form presented to the participating physicians who evaluated the patients. However, the main distinction between suicide attempt patients and appeal patients in this study was the suicidal intent. Given the similarity between suicide attempt and appeal patients observed in our study, terminology may focus on the overall level of intent rather than the presumed motivation implied by terms such as gesture or appeal. The terms "moderate to high suicide intent" versus "low or no suicide intent" might have been used instead.

## Conclusions

The present study demonstrated considerable similarities between suicide attempt patients and those who have not made suicide attempts regarding lack of social integration, substance use, previous suicide attempts and previous or current psychiatric treatment. Suicide attempt patients and appeal patients were generally quite similar, apart from the intention. Suicide attempt patients and appeal patients were more often referred to further treatment, while those in the substance use-related poisoning group were often discharged without such plans. The concordance between patients' and physicians' evaluations of intention was good. However, if intention is given too much weight, a large group of substance use-related poisoning patients seems to be excluded from further aftercare, despite their well-known risk of increased mortality and their substantial number of risk factors for suicidal behavior.

## Competing interests

The authors declare that they have no competing interests.

## Authors' contributions

MAB participated in the collection of data, performed the statistical analyses and drafted the manuscript. KEH participated in the design of the study and coordinated the collection of data. FH structured the data files and helped with the statistical analyses. KS coordinated the study at Oslo University Hospital Aker. PD coordinated the study at Lovisenberg Hospital. AO coordinated the study at Diakonhjemmet Hospital. DJ conceived the study and supervised the work. OE designed the present part of the study and supervised the work. All authors participated in revising the manuscript, and have read and approved the final version.

## Pre-publication history

The pre-publication history for this paper can be accessed here:

http://www.biomedcentral.com/1471-244X/10/58/prepub
